# Supplementary material for: Evaluation of an infection control protocol to limit COVID-19 at residential summer camps in 2021
Source: PLoS One. 2023 Nov 27;18(11):e0282560. doi: 10.1371/journal.pone.0282560 (PMC10681214; doi:10.1371/journal.pone.0282560)
Supplement: Attachment — Submitted filename: PLOS ONE-PEER REVIEW.docx [file pone.0282560.s001.docx]

**Introduction**

Well-written and flow of ideas is appropriate.

1. I recommend adding a paragraph about vaccine hesitancy (VH) among this age group, which made it even harder to accept vaccination especially at the beginning of rollout (May 10^th^, 2021). Indicate the relationship of VH and herd immunity.

Kindly add this as a reference: Musa S, Dergaa I, Abdulmalik MA, Ammar A, Chamari K, Saad HB. BNT162b2 COVID-19 Vaccine Hesitancy among Parents of 4023 Young Adolescents (12-15 Years) in Qatar. Vaccines (Basel). 2021 Sep 2;9(9):981. doi: 10.3390/vaccines9090981. PMID: 34579218; PMCID: PMC8473301.

2. Add another paragraph about the vulnerability to COVID-19 infection among different immune status, chronic disease, mental health, age, socioeconomic, nationality, gender, evidence from previous literature. [you need to link the interpretation of your study results with these factors, later in the discussion part].

**Methods**

**Under “Vaccination policy”**

1. Can you specify what do you mean by fully vaccinated? Two doses, three doses, type of validated vaccines, duration from vaccination (6 month/9 months/12 months).
2. How did you ensure that home test is carried out correctly to avoid measurement bias? Was there any training, or short video provided for participants?
3. Using the word ‘administered’ for the covid-19 test gives ambiguous meaning. You may replace throughout the manuscript with either “tested” or “undertaken”.

**Under “Camper characteristics”**

1. Kindly clarify the travel policy for those campers coming from Midwest region and outside US during the time of the airplane travel, in regard requirements of vaccination, quarantine and 24-48 hours negative PCR or rapid test requirements.
2. You have collected data on vaccination status, what about history of previous covid-19 infection and recovery? It is also an important information especially within first 6 months in terms of neutralizing antibodies and protection.

**Under “COVID-19 vaccination status”**

Kindly provide interpretation of the higher rates of vaccination among older age group. Previous literature in relation to vaccine hesitancy will assist including below citation:

Musa S, Dergaa I, Abdulmalik MA, Ammar A, Chamari K, Saad HB. BNT162b2 COVID-19 Vaccine Hesitancy among Parents of 4023 Young Adolescents (12-15 Years) in Qatar. Vaccines (Basel). 2021 Sep 2;9(9):981. doi: 10.3390/vaccines9090981. PMID: 34579218; PMCID: PMC8473301.

**Under “discussion”**

1. Will you explain how the staff were trained and prepared for receiving/handling mitigative procedures.
2. In addition, will you mention the successful adoption of the buddle system in the prevention and control of COVID-19 within other mass gathering for example among athletes/ world cup and compare the effectiveness of various interventions.

These citations will help you:

<https://www.researchgate.net/publication/358949547_Olympic_Games_in_COVID-19_times_lessons_learned_with_special_focus_on_the_upcoming_FIFA_World_Cup_Qatar_2022>

<https://www.researchgate.net/publication/358949547_Olympic_Games_in_COVID-19_times_lessons_learned_with_special_focus_on_the_upcoming_FIFA_World_Cup_Qatar_2022>

3. How the results of this study will aid in the planning within other settings, cohorts, and future emergencies. (add a section about implication on practice, policy and research).
