# Supplementary material for: Evaluation of an infection control protocol to limit COVID-19 at residential summer camps in 2021
Source: PLoS One. 2023 Nov 27;18(11):e0282560. doi: 10.1371/journal.pone.0282560 (PMC10681214; doi:10.1371/journal.pone.0282560)
Supplement: Attachment — Submitted filename: SINGSTMD - Rebuttal Letter .docx [file pone.0282560.s002.docx]

**Rebuttal Letter**

Dear Editors,

Thank you for reviewing our manuscript for publication. Please find our response to the reviewer’s comments below.

**Reviewer #1**

Introduction

1. I recommend adding a paragraph about vaccine hesitancy (VH) among this age group, which made it even harder to accept vaccination especially at the beginning of rollout (May 10th, 2021). Indicate the relationship of VH and herd immunity.
   Kindly add this as a reference: Musa S, Dergaa I, Abdulmalik MA, Ammar A, Chamari K, Saad HB. BNT162b2 COVID-19 Vaccine Hesitancy among Parents of 4023 Young Adolescents (12-15 Years) in Qatar. Vaccines (Basel). 2021 Sep 2;9(9):981. doi: 10.3390/vaccines9090981. PMID: 34579218; PMCID: PMC8473301.
   Thank you for your recommendation. We added a paragraph discussing vaccination rates and hesitancy to our introduction and included the provided references. Please see line 103.
2. Add another paragraph about the vulnerability to COVID-19 infection among different immune status, chronic disease, mental health, age, socioeconomic, nationality, gender, evidence from previous literature. [you need to link the interpretation of your study results with these factors, later in the discussion part].
   The authors felt that this was outside the scope of this paper. Our goal was to describe an infectious disease control protocol and the authors feel that an in-depth description of the epidemiology of COVID-19 vulnerabilities would not substantively contribute to the objectives of this paper.

Methods

1. Can you specify what do you mean by fully vaccinated? Two doses, three doses, type of validated vaccines, duration from vaccination (6 month/9 months/12 months).
   Our protocol specified fully vaccinated as two doses of either the Pfizer-BioNTech or Moderna vaccine. We have clarified this in line 130 under “Vaccination Policy.”
2. How did you ensure that home test is carried out correctly to avoid measurement bias? Was there any training, or short video provided for participants?
   Training for saliva collection was provided with the test package instructions. We have clarified this in line 138 under “Quarantine and Pre-Camp COVID-19 Testing Policies.”
3. Using the word ‘administered’ for the covid-19 test gives ambiguous meaning. You may replace throughout the manuscript with either “tested” or “undertaken.”
   We prefer to use the word administered because campers completed the rapid test under the guidance of a camp medic.
4. Kindly clarify the travel policy for those campers coming from Midwest region and outside US during the time of the airplane travel, in regard requirements of vaccination, quarantine and 24-48 hours negative PCR or rapid test requirements.
   All campers and their families were requested to wear a face mask and avoid public gatherings during the two weeks before arriving at camp, and a KN95 mask was provided for the camper to wear while traveling to and from camp, as described in “Quarantine and Pre-Camp COVID-19 Testing Policies.” Testing and quarantine policies did not vary based on the camper’s method of travel to camp.
5. You have collected data on vaccination status, what about history of previous covid-19 infection and recovery? It is also an important information especially within first 6 months in terms of neutralizing antibodies and protection.
   We agree that prior infection conveys protection. However, we did not collect this information from campers.
6. Kindly provide interpretation of the higher rates of vaccination among older age group. Previous literature in relation to vaccine hesitancy will assist including below citation: Musa S, Dergaa I, Abdulmalik MA, Ammar A, Chamari K, Saad HB. BNT162b2 COVID-19 Vaccine Hesitancy among Parents of 4023 Young Adolescents (12-15 Years) in Qatar. Vaccines (Basel). 2021 Sep 2;9(9):981. doi: 10.3390/vaccines9090981. PMID: 34579218; PMCID: PMC8473301.
   Thank you for this recommendation. We have included a discussion on vaccine hesitancy in the introduction (see line 105). A primary driver in this study was the FDA approval for different age groups.

Discussion

1. Will you explain how the staff were trained and prepared for receiving/handling mitigative procedures.
   Infirmary staff of the camps handled mitigating procedures. A description of their training is beyond the scope of this paper.
2. In addition, will you mention the successful adoption of the buddle system in the prevention and control of COVID-19 within other mass gathering for example among athletes/ world cup and compare the effectiveness of various interventions. These citations will help you: https://www.researchgate.net/publication/358949547_Olympic_Games_in_COVID-19_times_lessons_learned_with_special_focus_on_the_upcoming_FIFA_World_Cup_Qatar_2022
   We believe our research adds to the literature of the bubble system in mitigating disease spread. We have included a brief discussion on this topic to the discussion. A comparison of the effectiveness of various interventions was beyond the scope of this report.
3. How the results of this study will aid in the planning within other settings, cohorts, and future emergencies.
   Thank you for this suggestion. Please see the rewritten and expanded final paragraph.

**Reviewer #2**

General

1. The study had great potential to contribute to the evidence base to support the impact of safety precautions or protocols for infection control, especially for COVID-19 where there have been controversies, mixed with adherence issues. The meticulous attempt to implement and evaluate a well-crafted protocol is highly commendable.
   However, the paper is weak in providing strong statistical argument to support their findings. For a start, a measure of adherence to the protocol itself is an important piece in substantiating the results of the protocol’s effectiveness in preventing spread of COVID-19. Though this was pointed out by the authors themselves, along with other limitations that were mentioned including not conducting repeat tests at the end of the camping.
   Although not stated, this study was an interventional study of a quasi-experimental design, where there was no control group, but there was a form of pre-/post-intervention assessment of the outcome variable, COVID-19 test positivity.
   We appreciate these general comments. We agree that this research does constitute an experiment without control and, therefore, lacks rigorous statistical comparison. We provide reference to other camps, where applicable, to describe the incidence of COVID-19 without protective measures. Additionally, it is a limitation that we did not retest as campers left; however, we did closely monitor for COVID-19 symptoms. Therefore, we feel confident that if a camper or staff member was symptomatic for COVID-19, we would have identified them.

Methods

2. The components of the Protocol (that is the ‘intervention’) that was implemented were excellently described, however could be intentional in indicating them as subsumed under this intervention. The authors should consider this revision to display a sub-section under the Methods section. This could substitute for the table on the list of CDC-recommended procedures for camps that was displayed in the introduction section, which could simply be referenced.
   We appreciate the reviewer's comments. Given the frequently updated recommendations and changing knowledge since the beginning of the pandemic, we felt that a tabular representation of recommended procedures added to the generalizability and would enable readers to understand the state of science more easily in May 2021.
3. Pre-Camp COVID-19 Testing Policies - Authors could rephrase the statement that ‘parents were required to administer an at-home test via saliva collection’ as in essence it was an at-home sample collection that was done for a PCR test.
   This has been rephrased. Please see line 141.
4. COVID-19 Vaccination Status - Clarity should be provided on the numbers in parenthesis in Line 2.
   This clarification has been made. Please see line 212.
5. Symptomatic Testing for COVID-19 - The word “denied” in the sentence “Camper B tested positive while Camper C denied testing” should be clarified.
   This wording has been modified.

Discussion

6. It is not conventional to report additional even if pertinent findings in the Discussion section of original research papers. For instance, the authors reported the ‘one person on staff that self-reported a positive case after the camp ended’. Though it made for good reading and complimented the narrative on the study limitations, the Editor may wish to provide more guidance in keeping with the journal’s policy or style. Perhaps what was more glaring was the general absence of inferences on how the findings compare with other reports of evaluation of infection control protocols for COVID-19 among similar study populations. Authors should consider this revision to the discussion section.
   We have made this edit to the discussion. Thank you for your comments.
