# Supplementary material for: Evaluation of an infection control protocol to limit COVID-19 at residential summer camps in 2021
Source: PLoS One. 2023 Nov 27;18(11):e0282560. doi: 10.1371/journal.pone.0282560 (PMC10681214; doi:10.1371/journal.pone.0282560)
Supplement: Attachment — Submitted filename: 10.9-Rebuttal Letter - SINGSTMD.docx [file pone.0282560.s003.docx]

**Rebuttal Letter**

Dear Editors,

Thank you for reviewing our manuscript for publication. Please find our response to the reviewer’s comments below.

**Have the authors made all data underlying the findings in their manuscript fully available?**

The data is freely available through dryad with a link included in the data availability statement.

**Reviewer #1:**

Thank you for highlighting the raised concerns, kindly for some points, the author response was insufficient to meet the review requirements.
For instance, comment #2
Vulnerability due to coexisting conditions is an important parameter related to susceptibility and risk, hence the rate of infection which varies greatly between healthy and non-healthy individuals and so the prevention and control protocol.

Thank you for your feedback. We have added the following statement: “While healthy children are generally less likely to suffer severe symptoms of COVID-19 when compared to those with pre-existing medical conditions and the elderly, they are still vulnerable to potentially serious complications of illness and can spread the coronavirus throughout their communities. In children in the United States, the baseline rate of hospitalizations from COVID-19 was 8 in 100,000; however, these rates increase in children with obesity, chronic lung disease, and/or who are black [8].”

Under methods, comment #2
The review point asked to explain the travel policy, PCR testing, vaccination requirement and quarantine related to participants who travelled to the location of the camp. However, the authors have explained about the face mask! Strict requirements were introduced all over the world during the time of your study, even some countries have banned travels, hence, it is still important to clarify the raised points.

Thank you for your request for clarification. Our paper currently explains how campers arrived in a variety of transportation methods and any travel outside of the camp was only permitted for staff, which is described in the methods. We explained how PCR testing was performed at home and at camp for campers who were not fully vaccinated with 2 doses of either Pfizer or Moderna vaccines. We additionally describe our quarantine policy and cohorting.

Also for comment #1 discussion:
The authors response indicate that training of the main study intervention is not an important source of bias that could threaten the internal validity of the study. This is not out of scope concern, however, it is amongst the core requirement. Kindly describe how training of staff who applied the prevention strategies was carried out including the duration and numbers. How have you ensured that application of these strategies was on uniform and in place. (monitoring of intervention is an important indicator of outcome measure).

We have added this to the section on In-Camp Testing “All saliva collection was overseen by the medical staff or by staff members who were directly trained by the medical staff on how to collect saliva for PCR testing.” We also added that " Staff members were taught the in-camping cohorting guidelines prior to each camp session. Reminders were provided during each meal. Additionally, staff members were encouraged to correct campers and staff members who were not following masking or cohorting guidelines.”

We have eliminated the use of the word adherence from the article, because this was not an outcome directly measured. We appreciate your feedback.

The review aims to improve the quality, representation and coherence of the paper, therefore, it is important to response adequately to every raised concern. Thanks

**Reviewer #2:**

The paper presented the implementation of a COVID-19 prevention protocol that attempted a “closed ‘bubble’ system” and was evaluated for its effectiveness by monitoring pre- and post-intervention COVID-19 PCR test and symptomatic test positivity. Key study limitations in the study design and statistical analysis were also clearly identified and discussed. The authors have done well to address the concerns that were pointed out by the reviewers and resubmitted a revised manuscript.

However, a mistaken notion regarding ‘adherence’ to the protocol has not been addressed. On one hand, the authors rightly pointed out that “there was no measure of adherence to camp prevention guidelines [the protocol]”, which is an important variable in substantiating the results of the protocol’s effectiveness in preventing spread of COVID-19. The import of this is that adherence cannot be ‘reported’ as a result or an outcome, even if it was guaranteed during the implementation of the guidelines. However, in the Abstract, ‘adherence’ to the protocol was reported in the “results” as successful; it was also emphatically stated in the “conclusion” that adherence led to the study outcome, even though the level of adherence was not measured.

It is suggested that, without the benefit of a measure of adherence but instead a description of the steps taken, the statements regarding adherence should be rephrased in the paper, particularly in the abstract. For instance, the statements in lines 56, 61 & 63 that begin with “adherence to [the protocol]…” could be re-written as “implementation of [the protocol]...”

Thank you for your feedback. We agree that we cannot make statements about the efficacy of adherence to the protocol when adherence was not a measured outcome but was subsumed under the umbrella of COVID-19 cases. We have eliminated the word “adherence” from the article.
