# Supplementary material for: Evaluation of an infection control protocol to limit COVID-19 at residential summer camps in 2021
Source: PLoS One. 2023 Nov 27;18(11):e0282560. doi: 10.1371/journal.pone.0282560 (PMC10681214; doi:10.1371/journal.pone.0282560)
Supplement: Attachment — Submitted filename: 11.2-Rebuttal Letter - SINGSTMD.docx [file pone.0282560.s004.docx]

**Rebuttal Letter**

Dear Editors,

Thank you for reviewing our manuscript for publication. Please find our response to the reviewer’s comments below.

1. Please include very clear goal and objectives of your study at the end of the introduction section
2. Move the sub-sections "Camper's Characteristics" and "Covid-19 Vaccination Status" from the methods to the beginning of the results section.

Thank you for your recommendations. I have added the following statement to the end of the introduction section, “The purpose of this study was to assess the effectiveness of an infection control protocol developed to mitigate the spread of COVID-19 at two multi-week residential summer camps in 2021. The primary outcome of interest, therefore, is COVID-19 transmission rates within the summer camp.” Additionally, the camper characteristic and COVID-19 vaccination status sections have been moved to results.
